# Social isolation and intrinsic capacity among left-behind older adults in rural China: The chain mediating effect of perceived stress and health-promoting behavior

**DOI:** 10.3389/fpubh.2023.1155999

**Published:** 2023-03-24

**Authors:** Hong Su, Lijuan Xu, Hong Yu, Yuqiu Zhou, Yang Li

**Affiliations:** ^1^School of Nursing, University of Harbin Medical, Daqing Campus, Daqing, China; ^2^Medicine College, Lishui University, Lishui, China

**Keywords:** left-behind elderly, social isolation, perceived stress, health-promoting behavior, intrinsic capacity

## Abstract

**Background:**

Strengthening and maintaining the intrinsic capacity (IC) of the older adults is the key to preventing and delaying disability and promoting healthy aging.

**Objective:**

This study explores the relationship between social isolation, perceived stress, health promotion behavior, and IC of the left-behind older adults in rural areas and analyzes the chain mediating effect of perceived stress and health promotion behavior among social isolation and IC.

**Methods:**

From March 2021 to May 2022, a multi-stage sampling method was used to recruit participants from rural areas in Heilongjiang Province, China. The data were collected by the simplified version of the Lubben Social Network Scale, the Chinese Perceived Stress Scale, the Health-Promoting Lifestyle Profile-Chinese, and the Integrated Care for Older People (ICOPE) screening tool. We used the PROCESS macro for SPSS to determine the mediating effect of perceived stress and health-promoting behavior between social isolation and IC.

**Result:**

Social isolation score was positively correlated with health-promoting behavior (*r* = 0.78, *p* < 0.01) and IC (*r* = 0.67, *p* < 0.01), whereas it was negatively correlated with perceived stress (*r* = −0.63, *p* < 0.01). Perceived stress was negatively correlated with health-promoting behavior (*r* = −0.62, *p* < 0.01) and IC (*r* = −0.43, *p* < 0.01). The health-promoting behavior and IC were positively correlated (*r* = 0.56, *p* < 0.01). Bootstrapping values indicated that the chain-mediating effect of perceived stress and health-promoting behavior was statistically significant.

**Conclusion:**

Consequently, to improve the IC of the left-behind older adults, we should focus more on reducing the degree of social isolation of the left-behind older adults and improving their perceived stress and health-promoting behavior.

## 1. Introduction

By the end of 2016, there were around 240 million people over 60 years, accounting for 16.7 percent of the total population. The left-behind older adults accounted for 25.8% of the elder adults. It can be inferred that by the end of 2016, the population of the left-behind older adults in China was 59.88188 million (1, 2). The National Working Committee on Aging predicts that by 2030, there will be >200 million left-behind older adults people in rural areas (3). The left-behind older adults in rural areas refers to those with rural household registration, aged above 60, whose children under the same household registration go out for work for more than 6 months each year, and who have no one to support them (4). Human organ and tissue functions, as well as physical abilities, gradually decline with age. Some older people might also have impaired daily activities and even lose the ability to live independently. China's disabled and semi-disabled older adults had reached 40 million by the end of 2019, with rural disabled and semi-disabled older adults accounting for 64.5% (5). Based on this, it is inferred that nearly 10 million of the left-behind older adults are disabled. Due to the lack of family care capacity for children working outside, the limited economic capacity of rural families, and the insufficient supply of social services at home, providing disability care for the older adults left behind in rural areas face many challenges. Therefore, preventing the disability of rural left-behind older people is significant for reducing the burden of family care and realizing healthy aging.

Strengthening and maintaining the intrinsic capacity (IC) of the older adults is the key to preventing and delaying disability and promoting healthy aging. IC is a combination of all the physical and mental strength that an individual can use at any time and includes five areas: cognition, psychology, sensory function, vitality, and activity, which reflect the overall health status of the older adults (6). The decline in IC is common in the community and among hospitalized older people. Based on the National Population Representative Sample Survey results, the overall incidence of IC decline was 43%. The prevalence rate of IC decline in the rural older adults was 1.36 times higher than that of the urban older adults population (*p* < 0.001) (7). It has also been observed that older people living in rural areas have a higher rate of IC decline. According to the Convoy Model of Social Relations, the scale, quality, and function of an individual's social relationships change over time and play an important role in the mental and physical health of the older adults (8). For the left-behind older adults in rural areas, maintaining proper living conditions is extremely difficult. As they have very limited sources of income, they usually undertake heavy agricultural labor or “intergenerational education.” They sometimes may even encounter the double exclusion of material and spiritual support, thus, showing a state of “social isolation.” In a state of lack of social resources, social isolation can easily lead to a variety of adverse health outcomes for the older adults, which can significantly reduce their physical status, cognitive function, and mental health level, lead to declined IC, and increase their readmission as well as mortality rates. Social isolation can have serious effects on the quality of life and safety of the older adults (9).

Perceived stress refers to the process of perceptual evaluation of stress, i.e., individuals make an integrated assessment of external stressful events through the perceptual process involving three stages of perception: perceptual organization, judgment, and recognition (10). Several studies have shown that social isolation can indirectly affect the cognition, emotion, and physical activities of the older adults through perceived stress, leading to a decline in IC (11, 12). Yang et al. (10) examined the perception mechanism more precisely, i.e., after being stimulated by threatening events and negative factors in life, the psychological changes after subjective cognitive system evaluation usually showed symptoms of tension and out-of-control. Furthermore, it can potentially harm the individual's physiology, emotion, cognition, and behavior, which is a high-risk factor leading to a lower IC (13). It is suggested that social isolation can also lead to an increase in perceived stress, leading to a decline in IC.

Health-promoting behavior is a series of methods or actions that can improve an individual's health, such as physical exercise and stress management behavior. The emergence of social isolation may hinder the older adults from obtaining social support from available social networks and establishing health awareness, thus, affecting their health-promoting behavior, leading to less physical activity, anxiety, depression and decline in cognitive functions, weakening the IC of the rural left-behind older adults (14, 15). The main effect model emphasizes that good social relations positively impact an individual's health (16). Social network support can effectively regulate individual behavior and enhance the sense of control over one's life to maintain good health. Li et al. found that a high level of stress perception can hinder their health promotion behavior ability; the higher the perceived stress level, the lower the health promotion behavior ability (17).

However, there are few existing studies on the older adults left behind in rural areas to support this hypothesis. According to the main effect model, the relationship between social network relationships and overall health is established primarily through two mechanisms: first, the emotional support function of social support can effectively adjust the individual stress coping level and enhance the individual's ability to resist diseases, thereby maintaining overall health. Second, social network support can effectively regulate individual behavior and form more healthy behavior. The main effect model provides a theoretical basis for the psycho-behavioral path of social isolation on internal ability decline reported in this study (18). Therefore, this study aimed to explore the relationship and the mechanism of social isolation, perceived stress, health-promoting behavior, and intrinsic capacity in left-behind older adults in rural China, as these parameters play an important role in improving the IC and quality of life. This research proposes four hypotheses: (H1) Social isolation is related to IC; (H2) Perceived stress may play a mediating role between social isolation and IC; (H3) Health-promoting behavior may show a mediating function in social isolation and IC; and (H4) Perceived stress and health-promoting behavior may have a chain mediating effect between social isolation and IC.

## 2. Materials and methods

### 2.1. Participants

We chose Heilongjiang Province as the research site because it is located in northern China, in the cold circle of Siberian high pressure. Since winters are too cold and long to grow food, most young and middle-aged people go elsewhere to go work, leaving the older adults at home alone. And due to budgetary and time constraints, a nationwide survey could not be implemented, and we have mentioned this as a limitation in the discussion section. As this was a cross-sectional study, it used the multi-stage sampling method from March 2021 to May 2022, according to the 2020 GDP ranking of Heilongjiang Province, that displayed the division of economic levels of 13 regions in Heilongjiang Province into three squares: high, medium and low. In a cross-sectional study, the sample size was calculated using the random sample size formula: *n* = $u_{\alpha /2}^{2}\pi$*(1—*π*)/*δ^2^, n: estimated sample size; π: population rate, according to the literature review, social isolation accounted for 25% of the total older adults population (19); *u*_α/2_: the value corresponding to 95% confidence interval under the normal distribution curve, ***u***_**α/2**_ = 1.96, δ: allowable error. The maximum allowable error in this study was set at 5%. The sample size of the urban and rural surveys was determined to be 350 cases based on the non-response rate of 15% and to ensure the stability of the path model. In the first stage, researchers randomly selected three cities (Daqing, Jixi, and Qitaihe) from Heilongjiang Province based on their economic development. In the second stage, one township was randomly selected from each selected city, whereas in the third stage, two or three natural villages were randomly selected from each selected township. The cluster sampling method was used, and the older adults in all selected villages were included as study subjects. The inclusion criteria were as follows: (1) local permanent residents (registered in rural areas), (2) age ≥ 60 years old, (3) offspring who had been working outside for >6 months every year, (4) participants having clear consciousness and normal communication with investigators, and (5) participants providing informed consent and voluntary participation. We excluded all participants suffering from poor attention and listening skills, other sensory disorders, and serious mental and physical ailments.

### 2.2. Data collection

All participants were recruited from the wards after getting approval from the Ethics Committee of Harbin Medical University Daqing Campus. Data were collected through face-to-face interviews. First, the researchers explained the purpose of the study to all eligible participants, and those who agreed to take part signed a written informed consent form. The researchers explained the standardized instructions to all participants during the data collection process, and they then completed the questionnaire on their own. Each older adult completed the questionnaire in 30~40 min. The researchers clarified all points and concerns raised by the participants. In cases of incomplete self-filled questionnaires, the researcher read each questionnaire item and recorded their responses. All questionnaires are collected on the spot and checked for completion. Based on the inclusion rate, a total of 392 questionnaires were distributed in this survey, and 366 valid questionnaires were recovered, the response rate is 93.4%.

### 2.3. Measures

#### 2.3.1. The general information

General characteristics such as gender, age, education, marital status, monthly family income, and self-assessed health status that may have an impact on the IC in left-behind older adults in rural China were included based on a review of the literature.

#### 2.3.2. Social isolation

The simplified version of the Lubben Social Network Scale (LSNS), developed by Lubben, was used to assess social isolation (20). The scale included six items, further divided into two dimensions: family network and friend network. Each item was scored by a 6-point Likert scale (0–5), while the total scoring was done from 0 to 30. A score of < 12 indicated that the older adults were socially isolated. Furthermore, while scoring for family and friend networks, each dimension of the questionnaire < 6 points indicated family isolation and friend isolation, with a lower score suggesting a higher risk of social isolation. The Cronbach's α of the LSNS in this study was 0.794.

#### 2.3.3. Perceived stress

The Chinese Perceived Stress Scale (CPSS) used to assess perceived stress was developed by Cohen et al. (21) and translated by Yang and Huang (10). The scale consisted of 14 items, including two dimensions of tension and out of control. Using the Likert 5-grade scoring method, the score range was 0–56. The higher the score, the greater the psychological pressure perceived by the subjects. The Cronbach's α of the CPSS in this study was 0.847.

#### 2.3.4. Health-promoting behavior

Health-promoting behavior was measured by the Health-Promoting Lifestyle Profile-Chinese (HPLP-C). It was developed by Walker and translated by Chen. It included 40 items on a 4-point scale across six dimensions: nutrition behavior, health responsibility behavior, self-actualization behavior, social support behavior, exercise behavior, and stress management behavior (22, 23). The total score ranged from 40 to 160 points, with higher scores indicating a higher health-promoting behavior. The Cronbach's α of the HPLP-C in this study was 0.953.

#### 2.3.5. Intrinsic capacity

The assessment of IC was based on the WHO-published Integrated Care for Older People (ICOPE) screening tool (24), including the Mini-Mental State Examination (MMSE) (25), Short Physical Performance Battery Test (SPPB) (26), Mini-Nutritional Assessment Short Form (MNA-SF) (27), and Geriatric Depression Scale-15 (GDS-15) (28) and included following evaluations: (1) Cognition: two items about orientation and memory were spared from MMSE. (2) Locomotion: chair rise test assessed one part of the SPPB. (3) Vitality: weight loss and appetite loss were recorded according to MNA-SF. (4) Sensory: vision and hearing loss were measured using self-reported and validated questions. (5) Psychosocial: depressive symptoms were evaluated using two questions from the GDS-15. ICOPE screening tool contained nine dichotomous questions that were coded as 1 or 0. We derived a summary of IC scores by adding all the responses to nine dichotomous questions from five domains (possible range: 0–9). The higher the score, the better the capacity. The Cronbach's α of the CPSS in this study was 0.912.

### 2.4. Statistical analyses

SPSS22.0 statistical software was used for statistical analysis, including descriptive analysis, reliability coefficient, and Pearson's correlation analysis. Harman's single factor test was used to check the common method deviation of all the variables; Model 6 in the Hayes' SPSS PROCESS v3.5 macro program was used to analyze the mediating effect. A non-parametric Bootstrap method was used by re-sampling 5,000 times to verify the significance of the intermediary effect of perceived stress and health behavior in social isolation and IC.

## 3. Results

### 3.1. Common method deviation test

Harman's single-factor test was used to include all variables in this study in exploratory factor analysis. The results revealed that a total of 12 factors had characteristic roots >1. In contrast, the variation explanation rate of the first factor was 18.90%, which was less than the critical standard of 40%, indicating that there was no obvious common method bias problem in the study data.

### 3.2. Characteristics of the study population

A total of 366 rural left-behind older adults were included in this study, consisting of 166 males (45.4%) and 200 females (54.6%). Although the age ranged from 60 to 86 (71.0 ± 6.17) years old, the education level was mainly primary school or below. 40.8 and 19.4% of left-behind older people were isolated by family and friends, respectively, as shown in Table 1.

**Table 1:** Characteristics of the study population.

**Characteristics**	**Variables**	***N***	**%**
Gender	Female	200	54.6%
	Gender	166	45.4%
Age	60–70	163	44.5%
	70–80	150	41.0%
	>80	53	14.5%
Education level	Primary school and below	186	50.8%
	Secondary school	138	37.7%
	High school or above	42	11.5%
Marital status	Married	267	73.0%
	Single (never married, divorced, separated and widowed)	99	17.0%
Household monthly income	Low ( ≤ 1,000 rmb)	184	50.3%
	Middle (1,000–3,000 rmb)	132	36.1%
	High (>3,000 rmb)	50	13.6%
Social isolation	Family isolation	147	40.8%
	Friend isolation	70	19.4%
Self-reported perceived health status	Very poor	22	6.0%
	Poor	118	32.2%
	Fair	140	38.3%
	Good	77	21.0%
	Very good	9	2.5%

### 3.3. Relationship between social isolation, perceived stress, health-promoting behavior, and intrinsic capacity

In this study, the Lubben Social Network Scale was used to evaluate the social isolation level of the older adults. The lower the questionnaire score, the more serious the social isolation level. Social isolation was positively correlated with health-promoting behavior (*r* = 0.78, *p* < 0.01) and IC (*r* = 0.67, *p* < 0.01), whereas social isolation was negatively correlated with perceived stress (*r* = −0.63, *p* < 0.01). Perceived stress was negatively correlated with health-promoting behavior (*r* = −0.62, *p* < 0.01) and IC (*r* = −0.43, *p* < 0.01). Finally, there was a correlation between health-promoting behavior and IC (*r* = 0.56, *p* < 0.01, Table 2). It offers preliminary support for further research into the hypothesis.

**Table 2:** Statistical description and related analysis results.

	**M**	**SD**	**1**	**2**	**3**	**4**
1	20.26	3.30	1			
2	24.44	3.60	−0.63^**^	1		
3	103.77	21.18	0.77^**^	−0.62^**^	1	
4	5.49	1.53	0.68^**^	−0.54^**^	0.61^**^	1

1 = social isolation; 2 = perceived stress; 3 = health promoting behavior; 4 = intrinsic capacity.

** *P* < 0.01.

### 3.4. The mediation of perceived stress and health-promoting behavior in the relationship between social isolation and intrinsic capacity

**Figure 1:**
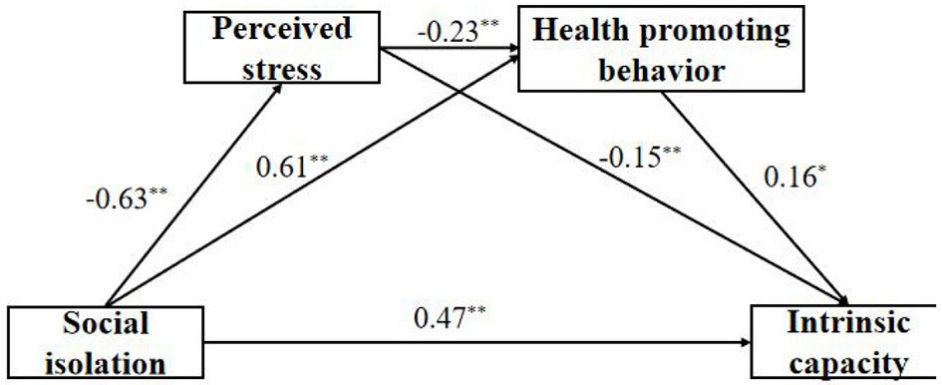
The chain mediating effect of perceived stress and health promoting behavior between health promoting behavior and intrinsic capacity. ^*^P < 0.05, ^**^P < 0.01.

According to Haye's SPSS macro program PROCESS, model 6 was used for regression analysis. The results showed that social isolation could significantly predict IC (β = 0.47, *p* < 0.01). Social isolation had a significant negative prediction of perceived stress (β = −0.63, *p* < 0.01) and a significant positive prediction of health-promoting behavior (β = 0.61, *p* < 0.01). Perceived stress had a significant negative predictive effect on the health-promoting behavior (β = −0.23, *p* < 0.01) and a significant negative predictive effect on IC (β = −0.15, *p* < 0.01). Finally, the health-promoting behavior positively predicted IC (β = 0.16, *p* < 0.05, Table 3). The preliminary judgment based on the structural equation model was that a perceived stress and health-promoting behavior mediation path existed, but the mediation effect (path coefficient of the product) required further verification. The non-parametric percentile bootstrap method was used to assess the mediation effect further. The results showed that the 95% CI corresponding to each path did not contain 0, which indicated the significance of the mediation effect, and established the chain mediation (Table 4). In line with our hypotheses, our results suggest that perceived stress and health-promoting behavior mediate the relationships between social isolation and intrinsic capacity. Figure 1 shows that social isolation affects intrinsic capacity directly among rural older adults; perceived stress affects intrinsic capacity directly among rural older adults; health promoting behavior affects intrinsic capacity directly among rural older adults; perceived stress and health-promoting behavior have a chain mediating effect between social isolation and IC.

**Table 3:** Regression analysis among variables in the chain intermediary model.

**Outcome variable**	**Predictor variable**	***R***	***R^2^***	**F**	**β**	***T***
Perceived stress	Social isolation	0.63	0.40	244.3^**^	−0.63	−15.63^**^
Health promoting behavior	Social isolation	0.79	0.63	304.40^**^	0.61	14.92^**^
	Perceived stress				−0.23	−5.74^**^
Intrinsic capacity	Social isolation	0.70	0.49	117.43^**^	0.47	7.57^**^
	Perceived stress				−0.15	−2.98^**^
	Health promoting behavior				0.16	2.54^*^

* *P* < 0.05,

** *P* < 0.01.

**Table 4:** Analysis of the mediating effect of perceived stress and health promoting behavior.

	**Effect size**	**Standard error**	**Boot CI LL**	**Boot CI UL**
Direct effect	0.465	0.061	0.000	0.344
Indirect effect 1	0.095	0.035	0.028	0.166
Indirect effect 2	0.096	0.038	0.021	0.170
Indirect effect 3	0.024	0.010	0.005	0.045
Total mediation effect	0.215	0.050	0.120	0.313

Indirect effect 1: social isolation → perceived stress → IC. Indirect effect 2: social isolation → health promoting behavior → IC. Indirect effect 3: social isolation → perceived stress → health promoting behavior → IC.

## 4. Discussion

The Cronbach's α of all questionnaires in this study ranged from 0.79 to 0.95, suggesting the ability to correctly identify the social and psychological characteristics of the older adults well. This study discovered a link between social isolation and IC. We assess social isolation using the Lubben Social Network Scale, so the lower the social isolation score, the greater the social isolation. Social isolation, as a state of loss of social network, is prone to a variety of adverse health outcomes in the older adults. These outcomes can significantly affect their cognitive, psychological, and physical activities, reduce their overall health level (intrinsic ability), and increase their readmissions and mortality (9). So the community service workers can understand the social network characteristics of the rural older adults and provide targeted services. For example, cultural and recreational activities should be provided to encourage the older adults to participate actively and make up for the loss of social connection caused by aging as much as possible.

In line with our hypotheses, our results suggest that perceived stress played a part in the mediating role between social isolation and IC in left-behind older adults in rural China. Because of a lack of interaction and contact with their children and family, social support, as well as physical and spiritual resources, the older adults in rural areas face multiple pressures, such as physiological degradation and psychological crisis. They are sensitive and paranoid about their environment, and their constant preoccupation with their health adds to the source of stress, which increases their perceived stress level. The “stress increase hypothesis” depicted that social isolation itself is a source of stress, triggering an inflammatory cascade of events, thereby weakening the IC of the left-behind older adults and leading to a variety of psychological and physical ailments (29). However, when an individual encounters setbacks and stress, it does not necessarily directly affect the individual itself; the induced perceived stress can produce a series of emotional and behavioral responses. The stress buffer mechanism exhibited that social networks effectively reduce individuals' negative responses to stressful events by affecting individuals' subjective evaluation of stress (30). The failure to relieve them in these stressful times can lead to a series of physiological, biochemical, and immune system changes, affecting the cognition, emotion, and physical activities of the older adults and leading to a declined IC (11, 12).

This study showed that health-promoting behavior partially mediated between social isolation and IC, which was consistent with previous study results (31). The social network, as an important environmental factor, significantly influences the health-promoting behavior of the older adults. The larger an individual's social network, and the closer their connection with social members, the easier it is to obtain some health-related information. The more they can regulate their behavior to promote health (32). A study by Robins et al. (15) showed that individual health promotion behavior is one of the direct factors in improving health outcomes. The older adults left behind in rural areas often suffer social isolation due to reduced social networking (15). The emergence of social isolation may also hinder the older adults from obtaining social support from social networks, and establishing health awareness, thus, affecting their health-promoting behavior and leading to adverse health outcomes. Lu et al. (33) showed that the health promotion of the older adults could promote the physical activity ability and the cognitive level of IC by encouraging good health behavior, such as weight control and moderate physical exercise. Therefore, it is of great practical significance to further improve the social network relationships of the left-behind older adults in rural areas, to improve their health behavior and the IC of the older adults, thus, preventing disability.

Finally, the social isolation level of left-behind older adults can also affect IC through the chain mediating effect of perceived stress and health-promoting behavior. Our study results supported the findings of Li et al. (17), i.e., perceived stress can affect healthy behavior. Stress levels are more closely related to emotional eating, eating control smoking, and drinking behaviors, while perceived stress is negatively related to health-promotion behavior (34). When an individual is stressed for a long time, the brain increases the individual's burden through “allostatic load,” which leads to systemic and functional insufficiency and initiates a series of adverse symptoms such as memory impairment, decreased concentration, and insomnia (8). It also affects the attention level toward an individual's health state and physical abilities, leading to a low healthy behavior ability. Perceived stress is one's own perception of external pressure, which is closely associated with poor physical and mental health. Social isolation can be regarded as a major risk factor that has been linked with increased awareness of stress among the left-behind older adults. Socially-induced stress affects the individuals' physiology, emotion, cognition, and mental behavior through the interplay of the nervous and endocrine systems. It can cause potential harm and a decline in their IC (35). As left-behind older adults in rural areas have been socially isolated for a long time, their social skills become poor, resulting in a lack of confidence while dealing with problems, eventually leading to a sense of tension and loss of control. Therefore, effective perceived stress management induces positive effects on IC. In contrast, the perceived stress management of rural left-behind older adults provides a new management path for the early prevention and treatment of disability.

Our research had some limitations. At first, while the mediation model was theoretically based, the cross-sectional study cannot fully infer the causal relationship between the variables. The use of longitudinal data in future studies could help to further investigate the conclusions of this study. Second, our study samples were all drawn from rural areas of Heilongjiang Province, limiting the generalizability of our findings. The diversity of the sample sources should be expanded in future studies. Thirdly, smaller samples cannot reflect the relationship between social isolation, perceived stress, health-promoting behavior, and intrinsic capacity to a greater extent. Because of the aforementioned limitations, the analysis of the results in our study should be reviewed cautiously.

## 5. Conclusion

The findings of the present study suggest that social isolation can directly affect intrinsic capacity and that perceived stress and health-promoting behavior mediate the relationship between social isolation and intrinsic capacity in left-behind older adults in rural China. This study's findings also add to the body of evidence highlighting the significant effects of social isolation on an individual's intrinsic capacity.

## Data availability statement

The data analyzed in this study is subject to the following licenses/restrictions: if necessary, data can be shared with the consent of the corresponding author. Requests to access these datasets should be directed to hltg210922@163.com.

## Ethics statement

The studies involving human participants were reviewed and approved by Harbin Medical University. The patients/participants provided their written informed consent to participate in this study.

## Author contributions

HS: conception and design of the study, drafting the article, and approval of the version to be published. LX: analysis and interpretation of data and drafting the article. HY and YL: contributed to data collection. YZ: interpretation of data, drafting the article, and approval of the version to be published. All authors contributed to the article and approved the submitted version.
